# Prognostic and clinical significance of modified glasgow prognostic score in pancreatic cancer: a meta-analysis of 4,629 patients

**DOI:** 10.18632/aging.202357

**Published:** 2021-01-06

**Authors:** Dongdong Wu, Xingmu Wang, Ge Shi, Honggang Sun, Guoxing Ge

**Affiliations:** 1 Clinical Laboratory Center, Shaoxing People's Hospital, Shaoxing Hospital of Zhejiang University, Shaoxing 312000, Zhejiang, China

**Keywords:** pancreatic cancer, modified Glasgow Prognostic Score, risk factors, meta-analysis, evidence-based medicine

## Abstract

In this study, we evaluated the association of modified Glasgow Prognostic Score (mGPS) with prognosis in pancreatic cancer (PC) by performing a meta-analysis. Potentially eligible studies were shortlisted by searching PubMed, Embase, Web of Science, Scopus, and the Cochrane Library. A total of 4,629 patients with PC from 25 studies were finally included in this meta-analysis. Meta-analyses were performed using a random-effects model or fixed-effect model according to heterogeneity. We pooled the hazard ratios (HRs) with 95% confidence intervals (CIs) to estimate the association between mGPS and overall survival (OS). The results showed that elevated mGPS correlated with poor OS in patients with PC (HR=1.92, 95% CI=1.60–2.30, p<0.002). In addition, subgroup analysis indicated that increased mGPS remained a significant prognostic factor irrespective of the study design, region, disease status, treatment, survival analysis, cancer type, study center, or the Newcastle-Ottawa Scale (NOS) score (all p<0.05). There was a significant correlation between higher mGPS and male gender (Odds ratio [OR]=1.30, 95% CI=1.01–1.67, p=0.038). Elevated pretreatment mGPS is a marker of poor prognosis in patients with PC. As an easily available and cost-effective inflammatory parameter, mGPS can serve as a promising tool for prognostication in PC.

## INTRODUCTION

Pancreatic cancer (PC) is one of the most aggressive malignant tumors with a very poor prognosis [1]. PC is currently the fourth leading cause of cancer-related deaths and is projected to rank second by 2030 [2, 3]. Notably, the majority of patients at the time of diagnosis have locally advanced or metastatic disease and are inoperable with a curative intent [4]. The prognostic indicators for PC include CA 19-9, SMAD4, microsatellite instability (MSI), and micro RNAs [5]. Although these markers have been used in clinical practice, the prognosis of PC patients has not substantially improved in the past decades. Moreover, obtaining adequate tumor tissue for analysis remains a major challenge for biomarker development in PC [5]. Therefore, there is an urgent need to identify more effective prognostic indicators that are also easily accessible for clinical use.

Cancer cells activate systemic inflammatory pathways that aid cancer progression by facilitating tumor cell proliferation, immune envision, and dissemination. A variety of inflammatory markers have attracted much attention as potential prognostic markers including C-reactive protein (CRP), neutrophil to lymphocyte ratio, and the modified Glasgow Prognostic Score (mGPS). The mGPS was established combining the levels of CRP and serum albumin. Some studies [6–15] have explored the prognostic efficiency of mGPS in PC, but the results remain conflicting. For example, some clinicians [9, 12, 14, 16] reported that elevated mGPS was a significant indicator of poor prognosis in patients with PC; however, other researchers found the association between mGPS and patient survival to be insignificant [7, 17, 18]. Therefore, we collected data from eligible studies and performed a meta-analysis to evaluate the prognostic role of mGPS in patients with PC.

## RESULTS

### Literature selection

**Figure 1:**
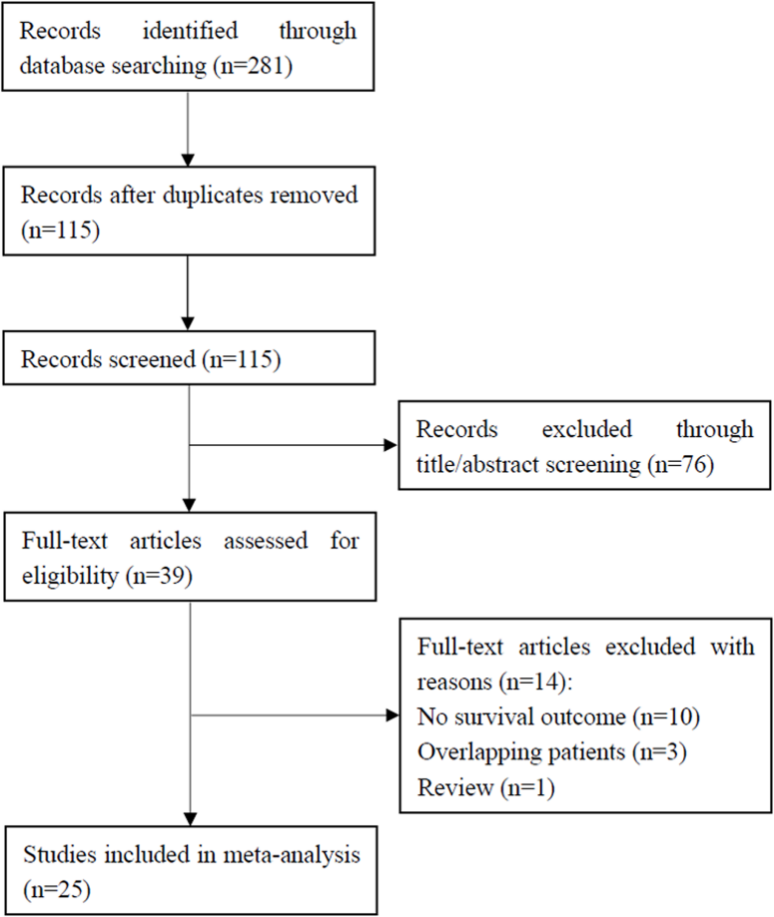
**Flowchart showing the study selection process.**

As shown in Figure 1, a preliminary literature search was carried out and 281 results were obtained. After removing 166 duplicates, 115 studies remained. Excluding 76 of the 115 studies by title and abstract review, 39 studies were shortlisted for full-text screening. Among them, 14 studies were excluded for the following reasons: 10 studies did not describe survival outcomes, 3 studies recruited overlapping patients, and 1 study was a review. Finally, a total of 25 studies [6–30] with 4,629 patients were included in this meta-analysis.

### Study characteristics

The basic characteristics of the 25 included studies are summarized in Supplementary Table 1. All studies reported the association between mGPS and OS in patients with PC. The total sample size was 4,629, with individual studies having a sample size ranging from 38 to 807. Three studies [8, 9, 20] were prospective and 22 studies [6, 7, 10–19, 21–30] were of retrospective design. Two of the included studies were conference abstracts [8, 9], and 23 studies were full-text articles [6, 7, 10–30]. Thirteen studies [6–10, 13, 14, 17, 20–22, 27, 29] recruited patients with pancreatic ductal adenocarcinoma (PDAC) and 12 studies [11, 12, 15, 16, 18, 19, 23–26, 28, 30] enrolled PC patients. Hazard ratios (HRs) and 95% confidence intervals (CIs) of multivariate analysis (MVA) were extracted from 19 studies [6–9, 11, 12, 14–16, 18, 21, 23–30] and 6 studies [10, 13, 17, 19, 20, 22] provided univariate analysis (UVA) HRs and 95% CIs. The Newcastle–Ottawa Scale (NOS) scores of all eligible studies were ≥6, indicating that all included studies were high-quality studies.

### Prognostic role of mGPS in OS and subgroup analysis

**Figure 2:**
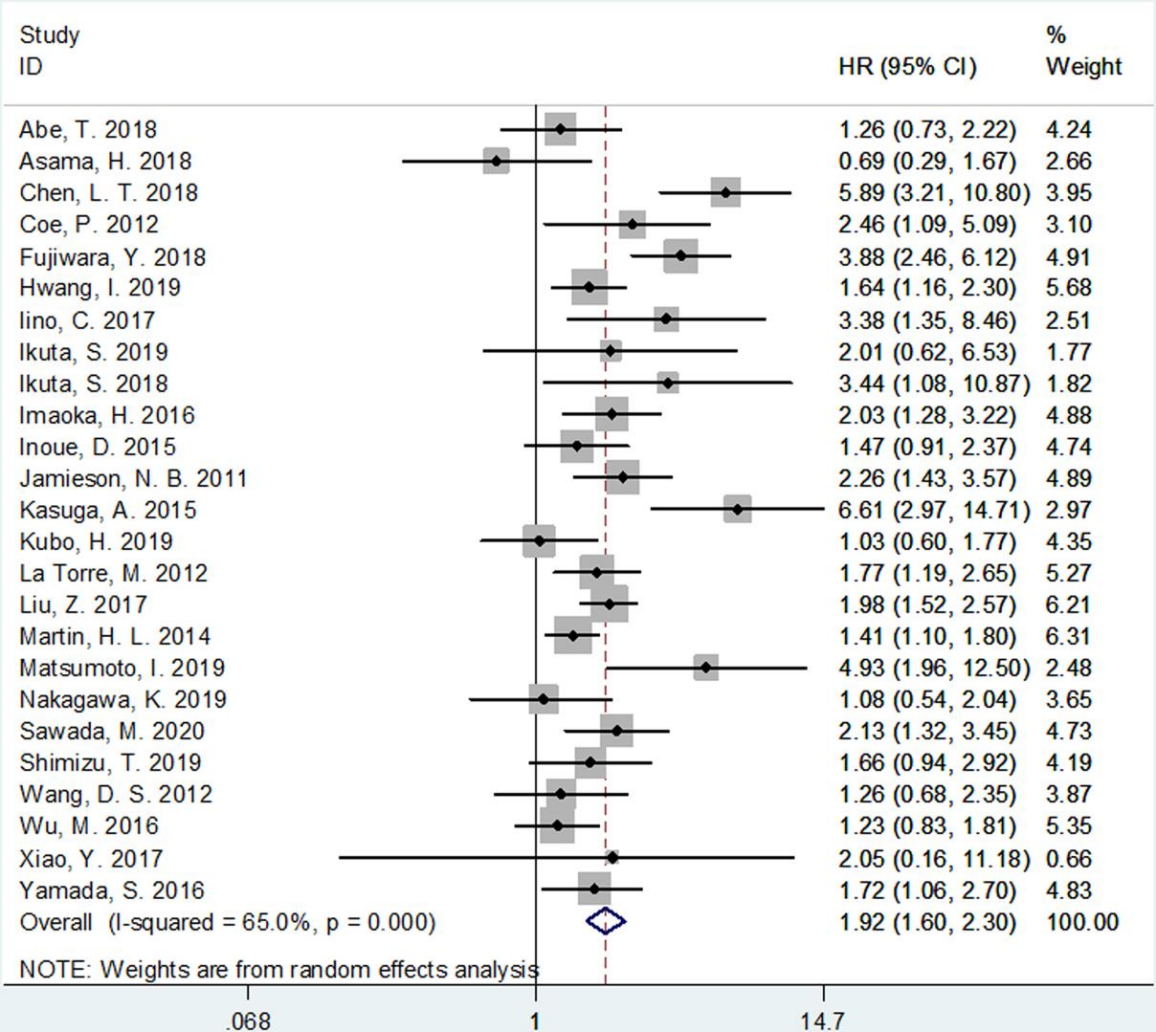
**Meta-analysis of impact of mGPS on overall survival in patients with pancreatic cancer.**

The correlation between mGPS and OS was investigated in all the 25 included studies [6–30]. As shown in Figure 2 and Table 1, because of significant heterogeneity (*I*^2^=65%, P<0.001), a random-effects model (REM) was used. It was shown that elevated mGPS is associated with poor OS (HR=1.92, 95% CI=1.60–2.30, p<0.002; Figure 2 and Table 1). Then, we conducted subgroup analysis; as detailed in Table 1. The pooled data indicated that increased mGPS remained a significant prognostic factor irrespective of the study design, region, disease status, treatment, survival analysis, cancer type, study center, or NOS score, with p<0.05 in all the above-mentioned subgroups. Regarding subgroups of tumor node metastasis (TNM) stage, mGPS was found to be a significant prognostic indicator in patients with stages I–III, III–IV, IV, I–II, and I–IV (Table 1).

**Table 1:** Subgroup analysis of the prognostic role of mGPS for OS in PC.

**Factors**	**Studies (n)**	**HR (95%CI)**	**p**	**Heterogeneity**		**Effects model**
				***I*^2^(%)**	**P**	
Overall survival						
Total	25	1.92(1.60-2.30)	<0.002	65	<0.001	REM
Study design						
Retrospective	22	1.78(1.49-2.13)	<0.001	59.3	<0.001	REM
Prospective	3	3.19(1.71-5.95)	<0.001	69.1	0.039	REM
Region						
Asia	21	1.95(1.56-2.43)	<0.001	68.1	<0.001	REM
Non-Asia	4	1.65(1.37-1.99)	<0.001	33.9	0.209	FEM
Disease status						
Non-metastatic	6	2.37(1.84-3.06)	<0.001	47.8	0.088	FEM
Locally advanced/metastatic	3	1.42(1.16-1.74)	0.001	49.7	0.137	FEM
Recurrent/metastatic	5	2.01(1.58-2.54)	<0.001	76.4	0.002	REM
Metastatic	2	2.99(1.98-4.52)	<0.001	88.8	0.003	REM
Mixed	9	1.68(1.44-1.98)	<0.001	33.1	0.153	FEM
Treatment						
Surgery	8	2.10(1.72-2.55)	<0.001	40.8	0.106	FEM
Non-surgery	12	2.10(1.51-2.92)	<0.001	76.8	<0.001	REM
Mixed	5	1.70(1.41-2.04)	<0.001	37.7	0.170	FEM
Survival analysis						
MVA	19	1.91(1.54-2.38)	<0.001	64.7	<0.001	REM
UVA	6	1.96(1.38-2.77)	<0.001	68.3	0.007	REM
Cancer type						
PDAC	13	1.98(1.48-2.66)	<0.001	68.6	<0.001	REM
PC	12	1.83(1.47-2.28)	<0.001	58.9	0.005	REM
Study center						
Single center	23	1.78(1.51-2.09)	<0.001	54.9	0.001	REM
Multi-center	2	5.58(3.36-9.28)	<0.001	0	0.753	FEM
NOS score						
<7	2	3.94(1.68-9.24)	0.002	67.2	0.081	REM
≥7	23	1.80(1.52-2.14)	<0.001	58.4	<0.001	REM
TNM stage						
I-III	4	1.76(0.81-3.83)	0.152	81.8	0.001	REM
III-IV	9	1.92(1.30-2.84)	0.001	73.0	<0.001	REM
IV	3	2.64(1.33-5.25)	0.006	84.6	0.002	REM
I-II	3	2.28(1.57-3.31)	<0.001	0	0.960	FEM
I-IV	6	1.79(1.52-2.11)	<0.001	0	0.736	FEM

PDAC: pancreatic ductal adenocarcinoma; PC: pancreatic cancer; OS: overall survival; MVA: multivariate analysis; UVA: univariate analysis; NOS: Newcastle-Ottawa Scale; mGPS: modified Glasgow Prognostic Score; REM: random-effects model; FEM: fixed-effects model; HR: hazard ratio; CI: confidence interval; TNM: Tumor Node Metastasis.

### Correlation between mGPS and clinical factors

**Figure 3:**
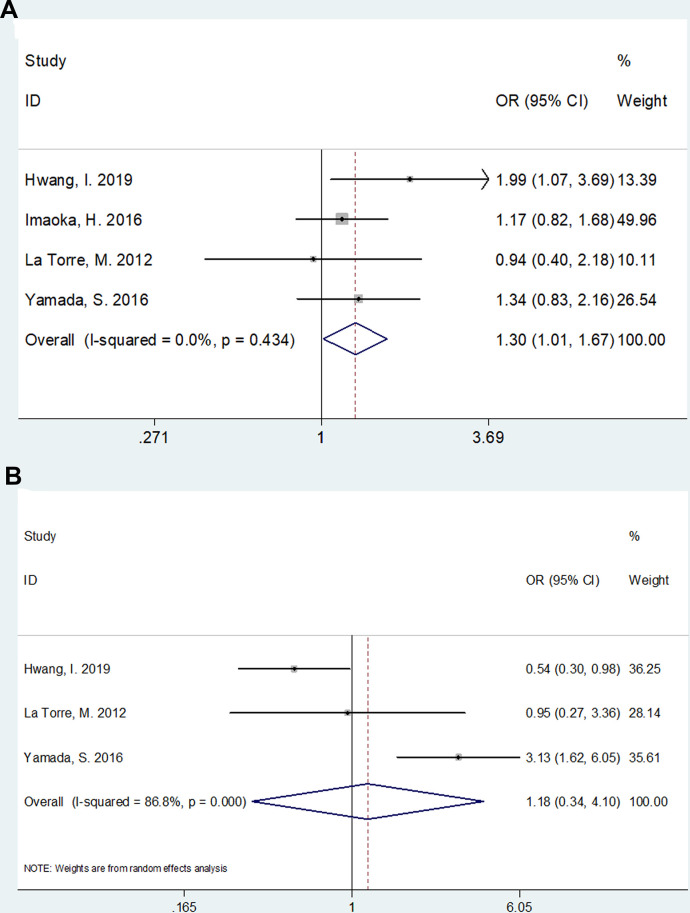
**The association between mGPS and clinical factors in pancreatic cancer.** (**A**) mGPS and sex (male vs female); (**B**) mGPS and tumor location (head vs body/tail).

The association between mGPS and clinical factors including sex (male vs. female) and tumor location (head vs. body/tail) was analyzed based on data from 4 [11, 15, 21, 30] and 3 studies [11, 21, 30], respectively. As shown in Figure 3, there was a significant correlation between higher mGPS and male gender (Odds ratio [OR]=1.30, 95% CI=1.01–1.67, p=0.038). However, the association between mGPS and tumor location was not significant (OR=1.18, 95% CI=0.34–4.10, p=0.792; Figure 3).

### Sensitivity analysis

**Figure 4:**
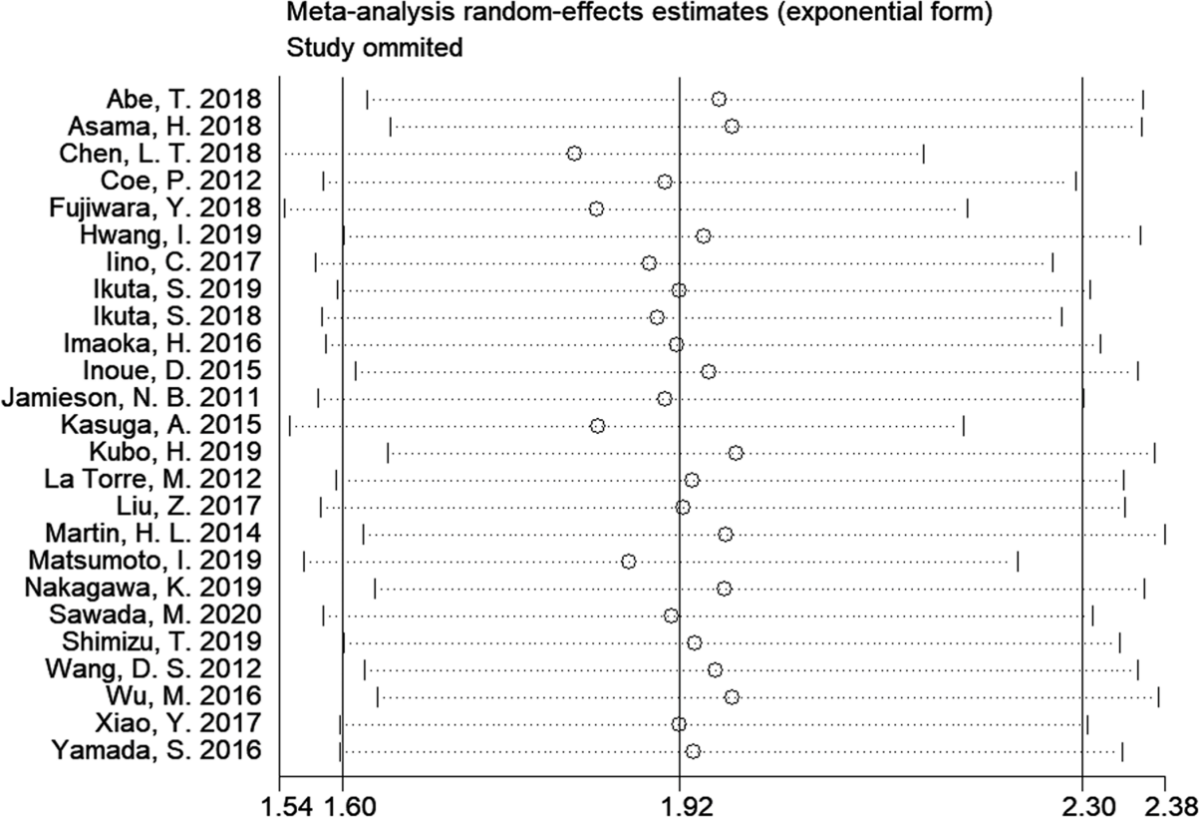
**Sensitivity analysis on the relationship of mGPS and overall survival in patients with pancreatic cancer.**

A sensitivity analysis was carried out by calculating the combined HR and 95% CI after omitting one study each time. As shown in Figure 4, no significant changes in the results were found by the omission of each study, suggesting that the results were robust.

### Publication bias

**Figure 5:**
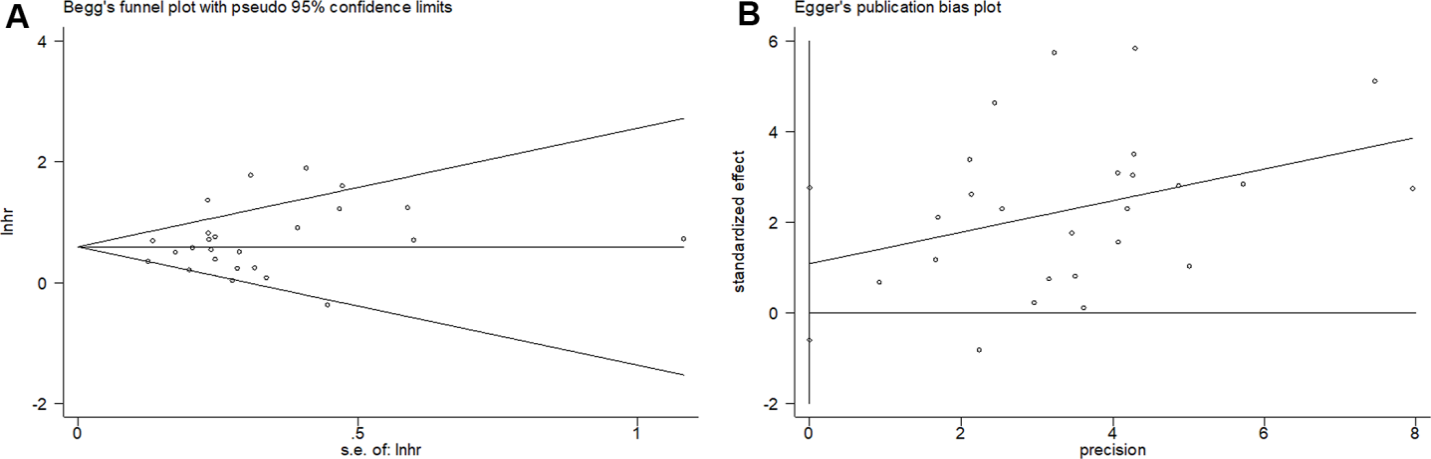
**Begg’s funnel plots and Egger’s publication bias plots for studies involved in the meta-analysis.** (**A**) Begg’s test of overall survival (p=0.388) and (**B**) Egger’s test of overall survival (p= 0.197).

As shown in Figure 5, the results from Begg’s funnel plot (p=0.388) and Egger’s test (p= 0.197) indicated that there was no significant publication bias in the current meta-analysis.

## DISCUSSION

In the present meta-analysis, we showed that a high mGPS was a prognostic indicator for poor survival in patients with PC. Moreover, the prognostic efficiency of mGPS was consistent and was not influenced by the study design, region, disease status, treatment, survival analysis, cancer type, study center, and NOS scores. In addition, the results suggested that male PC patients tend to have higher mGPSs, and therefore, have worse prognosis. Therefore, our meta-analysis indicates that mGPS could be applied as a reliable and cost-effective prognostic marker for PC.

Inflammatory responses play pivotal roles in tumor microenvironment, which can educate cancer cells to evade immune surveillance [31]. The mGPS is calculated based on the serum CRP and albumin levels. This score reflects both the inflammatory and the nutritional status of patients. CRP is a typical acute phase protein and is mainly produced by hepatocytes in response to inflammation, tissue damage, and infection [32]. Elevated levels of CRP have been reported as a prognostic factor for poor survival outcomes in a variety of cancers [32, 33]. Hypoalbuminemia is considered an indicator of malnutrition and cachexia, reflecting the poor physical condition of patients. Therefore, the elevation of mGPS could be common in patients with cancer and can serve as a prognostic marker for these patients.

Previous studies have explored the prognostic role of mGPS in various cancers using meta-analysis [34–39]. Nie et al. conducted a meta-analysis including 11 studies with 2,830 patients and showed that mGPS predicted poor OS and progression-free survival (PFS) in patients with gynecologic cancers [38]. Lu and colleagues also reported that patients with elevated mGPS were associated with poor OS and cancer-specific survival (CSS) in colorectal cancer [39]. In addition, a recent meta-analysis of 20 studies demonstrated that mGPS might be an independent prognostic factor in patients with urological cancers [36]. The present meta-analysis showed the prognostic effect of mGPS in patients with PC, which implies the potential use of mGPS as a common prognostic factor in cancer patients. In most subgroups of our meta-analysis, mGPS remained an effective prognostic indicator, except for patients with TNM stage I–III. This finding suggests that the prognostic power of mGPS could be greater in advanced cancer than in operable cancer [40].

Some limitations of the current meta-analysis require acknowledgment. First, most of the included studies were of retrospective design and the results were easily influenced by confounding factors. However, in the subgroup analysis stratified by survival analysis (Table 1), the results of both MVA and UVA showed a significant prognostic value of mGPS for pancreatic cancer. The data suggested that the survival analysis types did not influence the prognostic efficiency of mGPS. Further prospective studies with MVA of mGPS are still needed. Second, the recruited patients were in different TNM stages. As patients with advanced cancer may have higher mGPS, the diverse TNM stages may cause heterogeneity among studies. Third, a prognostic model for patients with PC could not be developed due to insufficient data of the included studies, compromising the originality of the current meta-analysis. We suggest that a specific prognostic model or nomogram incorporating mGPS should be explored for PC in future studies.

In conclusion, elevated pretreatment mGPS is a marker of poor prognosis in patients with PC. The prognostic efficiency was reliable across different subgroups. As an easily obtainable and cost-effective inflammatory parameter, mGPS can serve as a promising indicator for prognostication in PC.

## MATERIALS AND METHODS

### Study guidelines and ethical considerations

The current meta-analysis was carried out in accordance with the Preferred Reporting Items for Systematic Reviews and Meta-Analyses statement [41]. The ethical approval and patient consent were waived because all analyses were based on previously published articles.

### Search strategy

We retrieved potentially eligible studies by searching PubMed, Embase, Web of Science, Scopus, and the Cochrane Library. The search period was from inception to June 17 2020. Search terms used were the following free text words and medical subject heading (MeSH) terms: (pancreatic cancer OR pancreatic ductal adenocarcinoma OR pancreatic tumor) AND (mGPS OR modified Glasgow prognostic score OR C-reactive protein OR albumin) AND (prognosis OR prognostic OR survival OR outcome). Reference lists of literature were also manually examined for eligible studies. Two reviewers (D Wu and X Wang) searched the database independently, and all disagreements were resolved by discussion.

### Selection criteria

The following inclusion criteria were applied: (1) prospective and retrospective studies exploring the association between mGPS and OS in PC, with OS being calculated from the date of diagnosis to the last date of follow-up or death from any cause [27]; (2) mGPS scoring system is used as previously described: patients with an elevated CRP level (> 1 mg/dL) plus hypoalbuminemia (< 3.5 g/dL) are allocated a score of 2, patients with albumin ≥3.5 g/dL and CRP > 1 mg/dL or albumin < 3.5 g/dL and CRP ≤1 mg/dL are defined as a score of 1, and patients with albumin ≥3.5 g/dL and CRP ≤1 mg/dL are allocated a score of 0 [42]; (3) HRs of OS and their 95% CIs can be obtained; (4) if HRs and 95% CIs of both UVA and MVA are provided, the data of MVA are collected; otherwise, the results of UVA or MVA are collected if only one type of analysis is conducted; (5) full-text articles or meeting abstracts; (6) published in English language. Studies that did not meet the above inclusion criteria were not considered.

### Data extraction and quality assessment

Two authors (G Shi and H Sun) independently extracted data from the included studies, and all discrepancies were resolved by discussions with a senior investigator (G Ge). Data extracted from the literature included the first author’s name, publication year, geographical region, histological type, study design, sample size, endpoint, age, disease status, survival analysis, treatment, study center, TNM stage, source of HRs, and HRs and 95% CIs. The HRs and 95% CIs of mGPS 1–2 vs. 0 were extracted. If studies provided the HRs and 95% CIs of mGPS 1 and mGPS 2 as two groups, the two groups were combined and the HR of mGPS 1–2 vs. 0 was computed as previously described [35, 43]. The quality of the included studies was evaluated using the NOS (http://www.ohri.ca/programs/clinical_epidemiology/oxford.asp). The NOS consists of 3 sections: selection (0–4 points), comparability (0–2 points), and clinical outcomes (0–3 points). The maximum score of NOS is 9 and studies scoring 6–9 are regarded as high-quality studies.

### Statistical analysis

All statistical analyses were performed using Stata software version 12.0 (StataCorp LLC, College Station, TX, USA). For every study, HR and 95% CI were used to assess the relationship between mGPS and the prognosis of PC patients. The association between mGPS and clinical factors was analyzed using ORs and 95% CIs. HRs and 95% CIs were directly extracted from the articles or calculated from the Kaplan–Meier (K–M) curves according to Parmar’s method [44]. Heterogeneity across included studies was evaluated using Cochran’s Q tests and Higgins I-squared (*I*^2^) statistics. Either p<0.1 or *I*^2^ > 50% indicated the existence of significant heterogeneity, and the REM was applied for analysis. Otherwise, a fixed-effects model (FEM) was adopted. Subgroup analysis stratified by study design, region, disease status, treatment, survival analysis, cancer type, study center, NOS score, and TNM stage were performed to detect the source of heterogeneity. We also performed a sensitivity analysis to evaluate the validity of the combined results. Moreover, we used the Begg’s funnel plot and Egger’s test to evaluate publication bias. A p-value of less than 0.05 was considered statistically significant.

## Supplementary Material

Supplementary Table 1
