# Supplementary material for: Prognostic and clinical significance of modified glasgow prognostic score in pancreatic cancer: a meta-analysis of 4,629 patients
Source: Aging (Albany NY). 2021 Jan 6;13(1):1410–21. doi: 10.18632/aging.202357 (PMC7835027; doi:10.18632/aging.202357)
Supplement: Supplementary Table 1 [file aging-13-202357-s001.docx]

**Supplementary Table 1. Characteristics of the included studies.**

| Study | Year | Country  /region | Cancer type | Study design | Sample size | Endpoint | Age, years  Median(range) | Survival  analysis | Disease  status | Treatment | Study center | TNM stage | Source of HRs | HR (95%CI) | NOS score |
| --- | --- | --- | --- | --- | --- | --- | --- | --- | --- | --- | --- | --- | --- | --- | --- |
| Abe, T. | 2018 | Japan | PDAC | Retrospective | 329 | OS | 67 | MVA | Non-metastatic | Surgery | Single center | I-III | Reported | 1.258(0.726-2.220) | 7 |
| Asama, H. | 2018 | Japan | PDAC | Retrospective | 72 | OS | 63(42-85) | MVA | Mixed | Non-surgery | Single center | III-IV | Reported | 0.69(0.29-1.67) | 7 |
| Chen, L. T. | 2018 | Taiwan | PDAC | Prospective | 58 | OS | NR | MVA | Metastatic | Non-surgery | Multi-center | IV | Reported | 5.89(3.21-10.80) | 6 |
| Coe, P. | 2012 | UK | PDAC | Prospective | 94 | OS | NR | MVA | Non-metastatic | Surgery | Single center | I-II | Reported | 2.46(1.09-5.09) | 6 |
| Fujiwara, Y. | 2018 | Japan | PDAC | Retrospective | 188 | OS | 67(27-84) | UVA | Non-metastatic | Surgery | Single center | I-III | Reported | 3.880(2.460-6.121) | 7 |
| Hwang, I. | 2019 | Korea | PC | Retrospective | 203 | OS | 62(36-82) | MVA | Recurrent/metastatic | Non-surgery | Single center | IV | Reported | 1.64(1.16-2.30) | 8 |
| Iino, C. | 2017 | Japan | PC | Retrospective | 47 | OS | 75.8(66-80) | MVA | Locally advanced/metastatic | Non-surgery | Single center | III-IV | Reported | 3.38(1.35-8.46) | 8 |
| Ikuta, S. | 2019 | Japan | PDAC | Retrospective | 147 | OS | 68(33-86) | UVA | Non-metastatic | Surgery | Single center | I-II | Reported | 2.012(0.620-6.531) | 8 |
| Ikuta, S. | 2018 | Japan | PDAC | Retrospective | 38 | OS | 64(39-79) | MVA | Mixed | Non-surgery | Single center | III-IV | Reported | 3.436(1.079-10.870) | 8 |
| Imaoka, H. | 2016 | Japan | PC | Retrospective | 807 | OS | 65.6 | MVA | Mixed | Mixed | Single center | I-IV | Reported | 2.033(1.284-3.219) | 7 |
| Inoue, D. | 2015 | Japan | PC | Retrospective | 440 | OS | 67(32-88) | UVA | Mixed | Mixed | Single center | I-IV | Reported | 1.465(0.906-2.369) | 8 |
| Jamieson, N. B. | 2011 | UK | PDAC | Prospective | 135 | OS | NR | UVA | Non-metastatic | Surgery | Single center | I-II | Reported | 2.26(1.43-3.57) | 9 |
| Kasuga, A. | 2015 | Japan | PC | Retrospective | 61 | OS | 63(37-83) | MVA | Recurrent/metastatic | Non-surgery | Single center | III-IV | Reported | 6.605(2.965-14.709) | 9 |
| Kubo, H. | 2019 | Japan | PDAC | Retrospective | 119 | OS | 68 | UVA | Mixed | Mixed | Single center | I-III | Reported | 1.03(0.60-1.77) | 7 |
| La Torre, M. | 2012 | Italy | PDAC | Retrospective | 101 | OS | NR | MVA | Mixed | Surgery | Single center | I-IV | Reported | 1.775(1.187-2.653) | 7 |
| Liu, Z. | 2017 | China | PDAC | Retrospective | 386 | OS | 61(34-83) | UVA | Mixed | Mixed | Single center | I-IV | Reported | 1.98(1.52-2.57) | 8 |
| Martin, H. L. | 2014 | Australia | PC | Retrospective | 124 | OS | 68.5(35-90) | MVA | Locally advanced/metastatic | Non-surgery | Single center | III-IV | Reported | 1.41(1.10-1.80) | 8 |
| Matsumoto, I. | 2019 | Japan | PC | Retrospective | 66 | OS | 63(39-75) | MVA | Recurrent/metastatic | Non-surgery | Multi-center | III-IV | Reported | 4.93(1.96-12.5) | 9 |
| Nakagawa, K. | 2019 | Japan | PC | Retrospective | 172 | OS | NR | MVA | Recurrent/metastatic | Non-surgery | Single center | III-IV | Reported | 1.075(0.543-2.036) | 7 |
| Sawada, M. | 2020 | Japan | PC | Retrospective | 104 | OS | 63(37-77) | MVA | Recurrent/metastatic | Non-surgery | Single center | IV | Reported | 2.128(1.316-3.448) | 7 |
| Shimizu, T. | 2019 | Japan | PC | Retrospective | 83 | OS | NR | MVA | Metastatic | Non-surgery | Single center | III-IV | Estimated | 1.658(0.941-2.919) | 7 |
| Wang, D. S. | 2012 | China | PDAC | Retrospective | 177 | OS | NR | MVA | Mixed | Mixed | Single center | I-IV | Reported | 1.264(0.680-2.349) | 8 |
| Wu, M. | 2016 | China | PC | Retrospective | 233 | OS | 62(26-85) | MVA | Locally advanced/metastatic | Non-surgery | Single center | III-IV | Reported | 1.226(0.829-1.812) | 8 |
| Xiao, Y. | 2017 | China | PDAC | Retrospective | 66 | OS | 68.4 | MVA | Non-metastatic | Surgery | Single center | I-III | Reported | 2.05(0.16-11.18) | 7 |
| Yamada, S. | 2016 | Japan | PC | Retrospective | 379 | OS | 65 | MVA | Mixed | Surgery | Single center | I-IV | Reported | 1.723(1.062-2.702) | 8 |

PDAC: pancreatic ductal adenocarcinoma; PC: pancreatic cancer; OS: overall survival; MVA: multivariate analysis; UVA: univariate analysis; HR: hazard ratio; CI: confidence interval; NOS: Newcastle-Ottawa Scale.
